# The Spectrum of Movement Disorders in Anti-N-Methyl-D-Aspartate Receptor (NMDAR) Encephalitis Both in Children and Adults: An Experience From a Single Tertiary Care Center

**DOI:** 10.7759/cureus.20376

**Published:** 2021-12-13

**Authors:** Ayaz Ul Haq, Danish Nabi, Mehtab Alam, Samina A Ullah

**Affiliations:** 1 Neurology, Lady Reading Hospital, Peshawar, PAK; 2 Medicine, Khalifa Gul Nawaz Teaching Hospital, Bannu, PAK

**Keywords:** insomnia, dysautonomia, psychotic disorders, cognition, rituximab, hypokinesia, hyperkinesis, seizures, movement disorders, anti-n-methyl-d-aspartate receptor encephalitis

## Abstract

Anti-N-methyl-D-aspartate receptor (NMDAR) encephalitis is a form of autoimmune encephalitis. The characteristic clinical features include seizure, psychosis-like symptoms, abnormal movements, and autonomic disturbances. Patients with anti-NMDAR encephalitis can present with various types of movement disorders. Typically, the movement disorders start following intervals of psychiatric and prodromal manifestations in young adults; however, in children, these might be an early presentation of anti-NMDAR encephalitis. The disease is under-recognized and underdiagnosed in Pakistan. Early recognition of the disease is important to commence timely treatment leading to a better prognosis. Here we present a collection of anti-NMDAR encephalitis patients, specifically focussing on the different types of movement disorders and the differences in clinical manifestations between children and adults.

## Introduction

Anti-N-methyl-D-aspartate receptor (NMDAR) encephalitis is regarded as one of the most common types of autoimmune encephalitis [1]. Anti-NMDAR encephalitis is now more appreciated as a cause of treatable encephalitis, which affects children and young adults [2,3]. Young women are predominantly affected, either with or without ovarian teratoma. Early commencement of immunotherapy has an impact on the management of the disease and its outcomes [4]. Previously, “encephalitis lethargica” was the term used for encephalitis patients who presented with hypokinetic movement disorders along with insomnia and psychiatric manifestations but in recent years, most of them were realized to have anti-NMDAR encephalitis [5].

Autoimmune encephalitis, especially anti-NMDAR encephalitis is associated with various movement disorders [6,7]. The movement disorder can be an early feature of the disease [8]. Adult patients with anti-NMDAR encephalitis, before developing the typical movement disorders generally present with non-specific psychiatric symptoms. While in children, movement disorder and seizures may be the initial presenting complaints of anti-NMDAR encephalitis patients [9-11].

The clinical presentation of psychotic symptoms, seizures, abnormal movements, and autonomic disturbances is regarded as highly characteristic of NMDA-receptor antibodies in both female and male patients [12]. Clinical phenomenology can aid in this differentiation during the acute and early stages of autoimmune encephalitis, before a more characteristic clinical feature has developed or the results of antibody testing or imaging are available [6].

The phenomenology of the movement disorders associated with anti-NMDAR encephalitis still lacks consensus. Its clearer description will facilitate confident recognition and enable ensuing earlier immunotherapy in anti-NMDAR encephalitis [8]. The movement disorders in patients with ovarian tumors are much resistant to treatment [1].

The basic aim of this study is to characterize the different types of movement disorders and find the differentiating features between the children and adult patients diagnosed with anti-NMDAR encephalitis, which can help in early diagnosis.

## Materials and methods

After approval from Lady Reading Hospital Medical Teaching Institution Ethical Review Board with approval no. 50/LRH/MTI, a total of 17 patients (12 female and five male) were enrolled in this observational study. The study was conducted in the neurology unit of Lady Reading Hospital Peshawar from 18/05/2019 to 17/05/2021. An informed written consent (in Urdu) was taken from all participants. All those encephalitic patients were excluded from the study who had proven infective etiology for their presentations.

The age of study participants ranged from 15 months to 27 years with a median age of 8 years at symptoms onset. Among these patients, 10 were less than 12 years of age (median age of 6 years and mean age of 5.6 years) whilst seven patients were more than 12 years of age (median age of 18 years and mode of 14 years).

The diagnosis of anti-NMDAR encephalitis was made on the basis of history, electroencephalography (EEG), and cerebrospinal fluid (CSF) findings, ruling out other differential diagnosis and positive antibodies testing as proposed by international group diagnostic criteria for anti-NMDAR encephalitis. Data were analyzed through Statistical Package for the Social Sciences (SPSS) version 20 (IBM Corp., Armonk, NY). The percentages, frequency, mean, standard deviation (SD), and p-values were calculated.

## Results

Features of movement disorder

**Figure 1:**
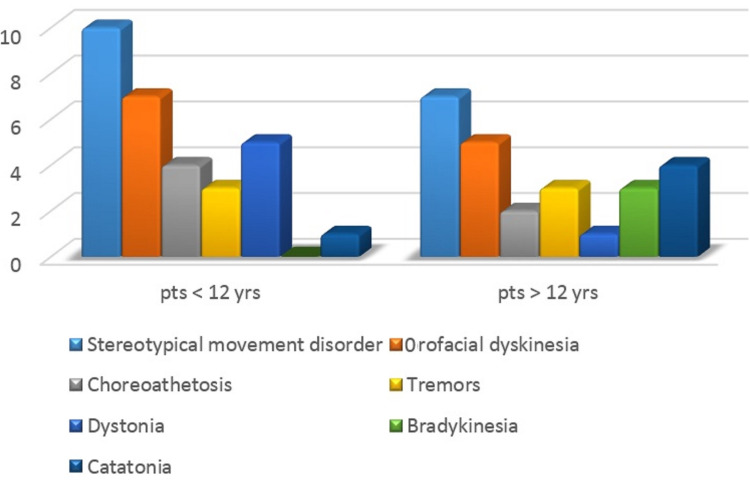
Comparison of various movement disorders pts=Patients

The characteristics of movement disorders in different age groups are summarized in Table 1 and Figure 1. All patients had more than one movement disorder recorded.

**Table 1:** Summary of the movement disorder in various age groups

Characteristics of movement disorders	Age < 12 years	Age > 12 years
Tremor	3	3
Orofacial dyskinesia	7	5
Stereotypical movement disorder	10	7
Catatonia	1	4
Bradykinesia	0	3
Dystonia	5	1
Choreathetosis	4	2

The most common movement disorder noted was stereotypical movement disorder defined as “involuntary, coordinated, repetitive, rhythmic, purposeless but seemingly purposeful or ritualistic movement, posture or utterance”. The stereotypy was either simple or complex stereotypies. The simple stereotypical movements included no-no or yes-yes movements of the head, cycling movements of legs, clapping hands, thrashing of the arm. This could progress to the rest of the body as well. One of the complex stereotypies noted was the patient moving in a circle with clapping hands and uttering sounds.

Orofacial dyskinesia was observed in 12 patients. Orofacial dyskinesia was characterized by involuntary and repetitive movements of face and mouth including grimacing, sucking, rolling, and protrusion of the tongue, jaw opening and closing. All patients were noted to have a stereotypical movement disorder. The most common movement disorder noted in children <12 years of age was stereotypical movement disorder followed by orofacial dyskinesia (71%). Among patients >12 years of age, orofacial dyskinesia was still the common movement disorder (71%) noted, followed by catatonia (57%). Comparing the two groups, the frequency of stereotypical movement disorder and orofacial dyskinesia was the same in both groups with no statistical difference. The hyperkinetic movements of dystonia, choreoathetosis were common in patients less than 12 years of age whilst the hypokinetic movements including catatonia and bradykinesia were more prevalent in patients more than 12 years of age. One of the patients presented with hypoventilation requiring ventilatory support.

Epileptic seizures

The majority of patients (12/17) suffered from seizures. Seven out of 12 patients presented with focal seizures with or without loss of awareness. Five patients presented with NORSE (new onset refractory status epilepticus). Two patients who were initially thought to have probable seizures based on the history provided actually had stereotypical movement disorder including clapping and turning around in circles.

Associated symptoms

**Figure 2:**
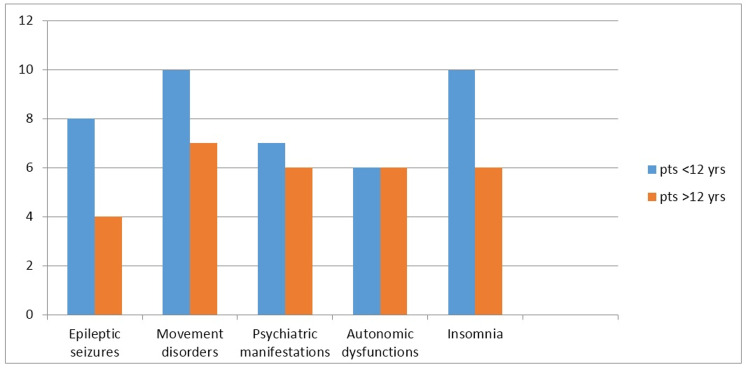
Age-wise distribution of all clinical manifestations pts=Patients

The associated clinical manifestations in different age groups are summarized in Figure 2. All the patients had experienced sleep disturbance during the initial phase of illness. Autonomic disturbance in form of fever, tachycardia, and at times fluctuating blood pressure was noted in most of the patients. Six patients (60%), less than 12 years of age whilst six patients (86%) more than 12 years of age had autonomic disturbance.

Immunomodulatory treatment

All patients were given IV methylprednisolone for five days. Six out of 17 patients had a good response to pulsed steroids. These patients were started on a steroid-sparing drug with weaning off oral steroids. Due to the financial constraints, some of the patients went on to have second-line immunotherapy i.e. rituximab instead of intravenous immune globulins (IVIGs). Four out of 17 patients required IVIGs. One of the patients had plasmapheresis. Six out of 17 patients received rituximab.

Thirteen patients had a good response and resumed normal life with two patients having persistent neuropsychological problems (aggression, paranoia, and lack of concentration) evident from poor academic performance and impaired social relationship after 18 months of their initial presentation. One of the patients presented and was diagnosed six months post-illness and therefore was left with occipital blindness and behavioral changes. Three out of 17 patients lost to follow-up.

## Discussion

The movement disorder is a frequent and emphasized feature of anti-NMDAR encephalitis but lacks an expert-based gold-standard description [2,3,6,12,13]. Patients suffering from anti-NMDAR encephalitis may exhibit a wide range and combination of movement disorders [2,6,14]. Orofacial dyskinesia, dystonia of face, trunk and limb, chorea, myoclonus, tremor, choreoathetosis, opsoclonus-myoclonus, and ataxia have been reported in approximately 86% of adults and 84% of children [2,7,15]. Another case series in children and adolescents showed that stereotyped movements (85%) and orofacial dyskinesia (45%) were the most common hyperkinetic movement disorder associated with anti-NMDAR encephalitis. The other associated hyperkinetic movements in this series were dystonia, chorea, and myorhythmia [16].

The comparison of clinical presentation between adults and children suffering from anti-NMDAR encephalitis shows a difference [17,18]. The adults mainly present with psychiatric and cognitive problems while features like movement disorders, refractory seizures, and behavioral changes are common in pediatric patients [18-20]. Among various associated movement disorders, patients older than 10 years of age mainly present with hypokinetic movement disorders like catatonia and bradykinesia, while hyperkinetic movements like choreoathetosis are more frequent in patients younger than 10 years of age [1].

Frequently convulsive and rarely non-convulsive seizures are manifestations of anti-NMDAR encephalitis [21]. About 2/3 of patients present with seizures [22]. As epilepsy is generally defined as a disorder of unprovoked recurrent seizures, patients with anti-NMDAR encephalitis have provoked (specific antibodies mediated) cause for their seizures; therefore, the term autoimmune epilepsy is avoided [22,23]. In one study of 75 patients with anti-NMDAR encephalitis, about 60% of the patients presented with either one or more than one type of seizure. The most common being is tonic-clonic followed by focal seizure with and without impaired awareness. In the same study, 1/3 of patients developed status epilepticus and 21% went into refractory status epilepticus [23]. New onset refractory status epilepticus (NORSE) may arise from anti-NMDAR and pose diagnostic and treatment challenges for immuno-therapy and refractory status epilepticus [24]. In another study, 81% of patients had seizures in their course of illness but at the end of two years follow up, all of them were seizure-free [25]. Data from these studies have shown that during the recovery phase, the gradual tapering of antiepileptic medications is safe and effective.

With regards to psychiatric symptoms, a retrospective study found that 2/3 (59%) patients presented with psychiatric symptoms. Among them 40% presented with visual or auditory hallucinations, 23% with acute schizoaffective episodes or depression, 8% with mania, and 6% with addictive or eating disorders [26]. Studies have also shown that these patients are quite neuroleptics sensitive [27].

Patients with anti-NMDAR encephalitis frequently show autonomic instability and respiratory dysfunction which necessitate their management in intensive care units. [26,28,29]. The most frequently encountered challenges in the management of such patients in intensive care units are to differentiate dyskinesia from true seizures, and nosocomial bacterial infection-induced fever from hypothermia secondary to autonomic instability of primary disease process [28]. Bradycardia, tachycardia, and elevation of blood pressure have also been seen. In the intensive care unit, these patients receive prolonged treatments with sedatives, empirical antibiotics, neuromuscular blockers, and anti-epileptics drugs [29-31].

## Conclusions

A variety of symptoms and presentations have been reported in patients with anti-NMDAR encephalitis. Movement disorders are typical but non-specific. More than one type of movement disorder is present in nearly all cases, a factor that poses a diagnostic challenge to the neurologist and can delay the start of appropriate treatment. Despite its small sample size, our study has shown that pediatric patients are more likely to develop hyperkinetic movement disorders, while hypokinetic movement disorders are more prevalent among adult patients. Early and accurate diagnosis of anti-NMDAR encephalitis and treatment initiation has significant long-term prognostic implications. Recognition of the movement disorders and other symptoms can help in the early diagnosis of anti-NMDAR encephalitis and its timely management.
